# Antioxidant and Anti-Senescence Effect of Metformin on Mouse Olfactory Ensheathing Cells (mOECs) May Be Associated with Increased Brain-Derived Neurotrophic Factor Levels—An Ex Vivo Study

**DOI:** 10.3390/ijms18040872

**Published:** 2017-04-20

**Authors:** Agnieszka Śmieszek, Zuzanna Stręk, Katarzyna Kornicka, Jakub Grzesiak, Christine Weiss, Krzysztof Marycz

**Affiliations:** 1 Department of Experimental Biology and Electron Microscope Facility, The Faculty of Biology and Animal Science, Wroclaw University of Environmental and Life Sciences, Norwida 25, 50-375 Wroclaw, Poland; zuzanna.strek@gmail.com (Z.S.); kornicka.katarzyna@gmail.com (K.K.); 2 Wroclaw Research Centre EIT+, Stablowicka 147, 54-066 Wroclaw, Poland; grzesiak.kuba@gmail.com (J.G.); krzysztof.marycz@up.wroc.pl (K.M.); 3 PferdePraxis Dr. Med. Vet. Daniel Weiss, Postmatte 14, CH-8807 Freienbach, Switzerland; d.weiss@horsedoc.ch

**Keywords:** metformin, olfactory ensheathing cells, brain-derived neurotrophic factor, senolytic, anti-oxidative

## Abstract

Metformin, the popular anti-diabetic drug was shown to exert multiple biological effects. The most recent metformin gained attention as an agent that mobilizes endogenous progenitor cells and enhances regenerative potential of organisms, for example by promoting neurogenesis. In the present study, we examined the role of metformin on mouse olfactory ensheathing cells (mOECs) derived from animals receiving metformin for eight weeks at a concentration equal to 2.8 mg/day. The mOECs expanded ex vivo were characterized in terms of their cellular phenotype, morphology, proliferative activity, viability and accumulation of oxidative stress factors. Moreover, we determined the mRNA and protein levels of brain-derived neurotrophic factor (BDNF), distinguishing the secretion of BDNF by mOECs in cultures and circulating serum levels of BDNF. The mOECs used in the experiment were glial fibrillary acidic protein (GFAP) and p75 neurotrophin receptor (p75^NTR^) positive and exhibited both astrocyte-like and non-myelin Schwann cell-like morphologies. Our results revealed that the proliferation of OECs derived from mice treated with metformin was lowered, when compared to control group. Simultaneously, we noted increased cell viability, reduced expression of markers associated with cellular senescence and a decreased amount of reactive oxygen species. We observed increased mRNA expression of BDNF and its down-stream genes. Obtained results indicate that metformin may exert antioxidant, anti-apoptotic and senolytic action on OECs expanded ex vivo.

## 1. Introduction

Currently, metformin (MET) is considered a first-line pharmacological treatment for type 2 diabetes (T2D). Metformin is prescribed to 150 million people each year, thus it is the most commonly used anti-diabetic orally administered drug [1]. Metformin has been also found to act as a pleiotropic agent exerting various beneficial effects besides its therapeutic action associated with lowering glucose level and improving insulin sensitivity [2,3]. Molecular pathways responsible for the metformin effect are still poorly understood; however, in vitro and in vivo studies clearly highlighted the fact that many metformin effects are mediated by a central regulator of energy homeostasis, i.e., AMP-activated protein kinase (AMPK) [4,5]. Recently, metformin gained attention as an effective next-generation drug, which can find application in regenerative medicine for the treatment of age-related diseases [6]. Instinctively, the anti-aging effect of metformin was correlated with increased antioxidant protection and reduction of DNA damage, all of which contribute to improving the regenerative potential of the body. The observation that metformin can promote adult neurogenesis and enhance spatial memory formation in mice has raised considerable interest related to the use of this drug in enhancing the potential of endogenous neural stem cells (NSCs) [7,8]. Moreover, studies of Labuzek et al. [9,10] showed that orally administered metformin rapidly crossed the blood-brain barrier and was distributed to various brain regions. It should be noted that metformin is one of only two orally administered anti-diabetic drugs that have been listed in the 16th World Health Organization Model List of Essential Medicines. Furthermore, therapeutic strategies include metformin administration for the treatment of Alzheimer’s disease [3,11,12]. Neuroprotective effects of metformin have been also confirmed by a recent study of Chen et al. [13] who investigated the effects of anti-diabetic drugs on hippocampal synaptic plasticity using mice models. These authors showed that metformin could exert anti-apoptotic effects by decreasing the ratio of caspase-3 fragment/procaspase-3 and increasing the ratio of Bcl-2/Bax in the hippocampus. A metformin pro-survival effect also involves its influence on mitochondrial biogenesis; for example, it was shown that metformin inhibited mitochondrial damage via an AMP-activated protein kinase-dependent pathway in neuronal cells [14,15]. Due to the fact that the functional and structural mitochondrial defects contribute to the pathogenesis of neurodegenerative diseases, including Alzheimer’s and Parkinson’s disease, it seems that metformin could ameliorate the plasticity of neuronal cells by modulating mitochondrial biogenesis and affecting the clearance of mitochondrial reactive oxygen species (ROS) [16,17]. Cellular ROS production is a common hallmark of apoptosis and senescence processes [18]. The most recent study of Chen et al. [19] showed that metformin attenuated cellular apoptosis and senescence induced in nucleus pulposus cells by tert-butyl hydroperoxide, thus metformin may be considered as a senolytic drug. The role of senescent cells in neurodegeneration and cognitive dysfunction has been previously shown [18,20]. Moreover, the increased risk of cognitive impairment in diabetes is also associated with senescence in the brain, however—not only neurons, but also infiltrating or glial cells [21]. Glial cells (i.e., astrocytes, oligodendrocytes and microglia) normally provide structural, metabolic and trophic support to neurons, thereby contributing to brain homeostasis [22]. Mansour et al. showed that cultured astrocytes from the brains of ageing rats were positive for the senescence-associated beta-galactosidase (SA-Bgal) and had reduced ability to support the survival of co-cultured neurons [23]. Recently, much attention has been paid to another type of glial cells, i.e., olfactory ensheathing cells (OECs), as a great candidate for the transplant-mediated repair of central nervous system lesions, especially in spinal cord injuries [24,25,26]. The major advantages of OEC application in regenerative medicine are the benefits of their autologous transplantation, that allow for avoiding immune rejection and, in turn, the immunosuppression, which could raise further risks and compromise the effectiveness of the transplanted cells [27]. For example, the studies of Tabakow et al. make a great contribution in this direction, showing that spinal cord injuries can be treated with autologous human OECs [28]. Additionally, the potential use of olfactory-derived cells is considered in the research on neurodegenerative disease treatment, as their biology may reflect pathological changes in the brain [29]. OECs are characterized as an antigenically and morphologically heterogeneous cell population that expresses glial fibrillary acidic protein (GFAP) and p75 neurotrophin receptor (p75^NTR^); this fact led to the suggestion that they resembled astrocytes and non-myelin forming Schwann cells [24]. As previously shown, metformin can enhance olfactory neurogenesis, resulting in a significantly increased number of BrdU-positive and NeuN-positive olfactory neurons in the granule cell layer [7].

Recent evidence demonstrated that exogenously applied brain-derived neurotrophic factor (BDNF) promoted migration of cultured OECs, thus it could also contribute to their survival and synaptic plasticity [30]. However, more importantly, OECs cultured in vitro can secrete BDNF and other neurotrophic factors, such as nerve growth factor (NGF) [30,31,32,33]. Considering the available data regarding the negative influence of metformin on neurotropic factors [3] and other conflicting information about the adverse effects of metformin on the brain [34,35], it would be reasonable to investigate this dual effect on OEC cellular metabolism. Therefore, the aim of this study was to investigate the effect of metformin treatment on the cellular activity of mice olfactory ensheathing glial cells. We determined the effect of metformin administration on phenotype, proliferative capacity, viability and oxidative status of mouse olfactory ensheathing cells (mOECs). Additionally, we determined mRNA and protein levels of BDNF, thereby differentiating the secretion of BDNF by mOECs in cultures and circulating levels of BDNF.

## 2. Results and Discussion

### 2.1. Cultures of mOECs Derived Ex Vivo from Metformin-Treated and Untreated Animals Show Distinct Morphological and Phenotypical Features

**Figure 1:**
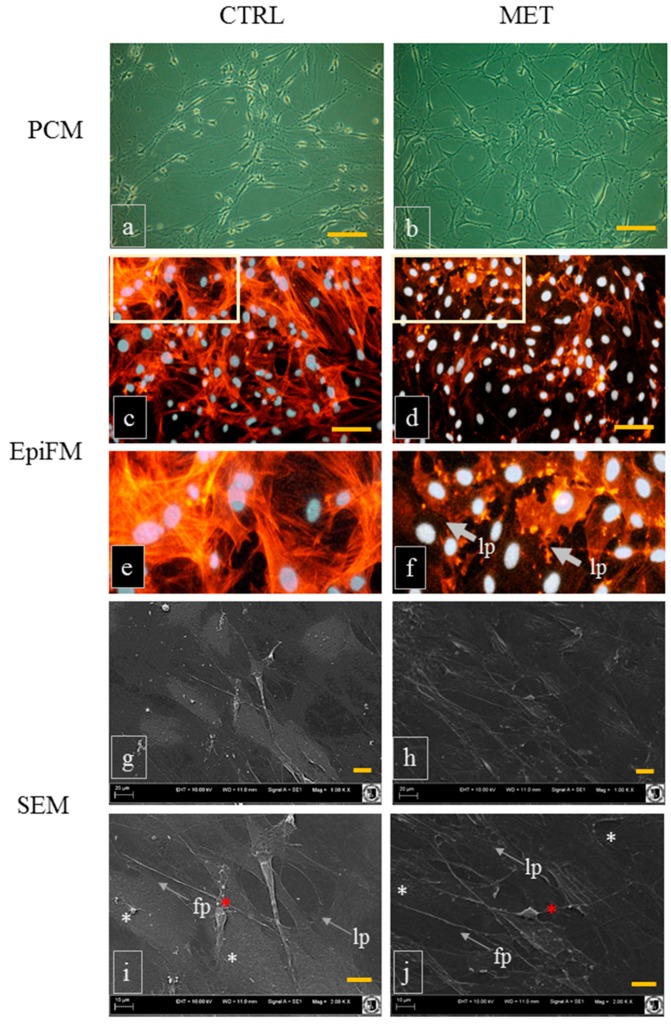
Morphological comparison of mouse olfactory ensheathing cells (mOECs) derived from animals of the control group (CTRL, non-treated with metformin) and receiving metformin (MET). The cultures were visualized using a phase contrast microscope (PCM; **a**,**b**, scale bar = 250 μm). Observations of nucleus location and cytoskeleton development were performed using an epifluorescent microscope (EpiFM; **c**–**f**). Cytoskeleton was stained using phalloidin and nuclei using DAPI i.e., 4′,6-diamidine-2′-phenylindole dihydrochloride (merged images, scale bar = 200 μm). SEM microphotographs were captured at a magnification of 1000-fold (**g**,**h**—scale bar = 20 μm) and 2000-fold (**i**,**j**—scale bar = 10 μm). Morphological features were indicated with respective abbreviations: fp: filopodia and lp: lamellipodia. Cells resembling astrocytes were marked with white asterisks, while cells with a spindle shape morphotype, resembling non-myelin Schwann cells, were marked with red asterisks.

**Figure 2:**
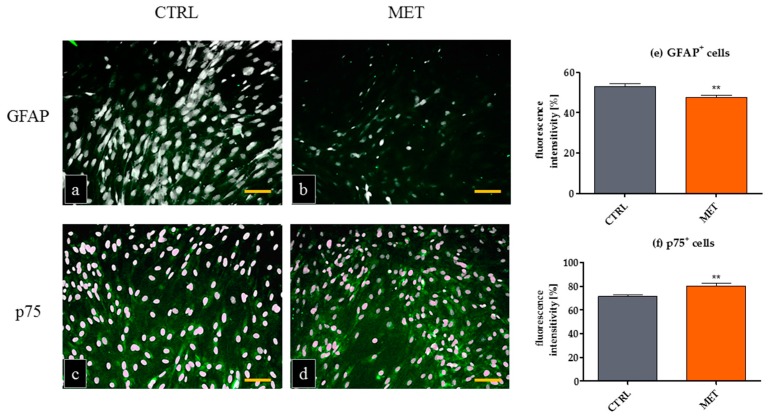
Immunofluorescence staining of mouse olfactory ensheathing cells (mOECs). The cells positive for glial fibrillary acidic protein—GFAP (**a**,**b**) and p75^NTR^ (**c**,**d**) are stained in green, nuclei are counterstained with DAPI. Epifluorescent microscope imaging was performed at a magnification of 100×, scale bar = 250 μm. The intensity of fluorescence for each marker (**e**,**f**) was measured using ImageJ software as described in the Materials and Methods section. The results are shown as means ± SD. ** *p* value < 0.01.

The evaluation of mOEC morphology revealed that the obtained cultures were morphologically very heterogeneous and displayed both astrocyte-like and Schwann cell-like morphologies (Figure 1). Such a dualistic character of OEC cultures in vitro has been observed previously [24,36]. Our analysis showed that the formation of lamellipodia was more visible in cultures of mOECs derived from MET animals. A study of Windus et al. [37] showed that the heterogeneity of OECs may be regulated by motile lamellipodial waves, which are crucial for OECs to recognize and interact with each other. In addition, time-lapse microscopy analysis revealed that these lamellipodial waves were highly mobile and determined the OECs migration [38]. The morphology of mOEC cultures evaluated using SEM showed that the cells isolated from MET mice had well developed cellular connections. The potential initiation of cell–cell contact by MET could contribute to the improved contact-mediated migration of OECs, which is crucial for their in vivo function within the olfactory system and migration far into the injury site [37,39]. The morphological diversity of OECs in cultures in vitro correlated with different antigen characteristics. GFAP is an intermediate filament associated with glial cells, a typical marker of OECs of flat astrocyte-like cell bodies. These cells express also an embryonic form of the neural cell adhesion molecule (E-NCAM), but the expression of p75^NTR^ is very low. In turn, OECs characterized by a spindle-shape morphotype express high levels of p75^NTR^, simultaneously demonstrating diffuse GFAP localization [36]. Our results suggest that metformin may also modulate antigen plasticity of mOECs, as evidenced by increased expression of p75^NTR^ and decreased expression of GFAP (Figure 2). The p75^NTR^ marker is highly expressed in glia during development and is induced after many types of injury. It may also promote cell survival and differentiation by interacting with Trk receptors. On the other hand, p75^NTR^ may also mediate cell death by interacting with sortilin as a co-receptor in response to proneurotrophins [40]. Thus, it seems that p75^NTR^ is a hallmark between the end of proliferation and the beginning of differentiation in a variety of neuronal subpopulations [41]. This also highlights a crucial aspect of cellular or environmental context of metformin action on mOECs.

### 2.2. Metformin Administration in Mice Affects the Proliferative Activity of mOECs *Ex Vivo*

**Figure 3:**
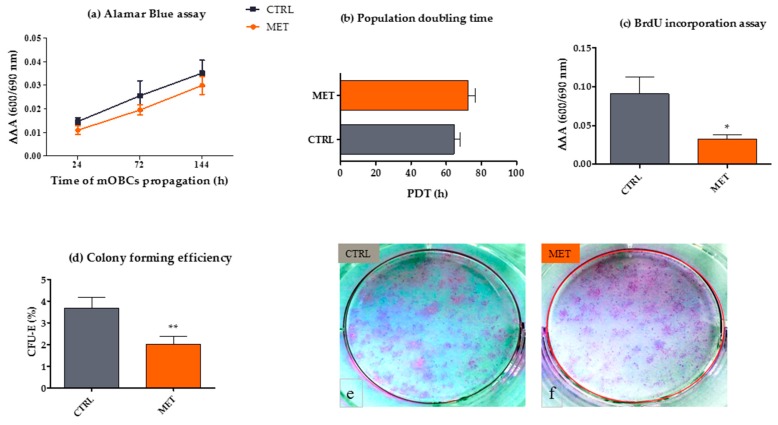
The analysis of proliferative activity of mouse olfactory ensheathing cells (mOECs) cultured ex vivo, derived from control animals (CTRL) and those receiving metformin (MET). Metabolic activity was assessed using Alamar Blue assay after 24, 72 and 144 h of propagation. The results of Alamar Blue assay are shown as ΔΔA value, expressing the difference in absorbance of the supernatants at 600 and 690 nm, including the absorbance of blank samples. Statistical analysis showed no significant difference in metabolic activity of mOECs derived from CTRL and MET animals (**a**); The analysis of population doubling time indicated lowered proliferative potential of OECs derived from MET mice; however, no statistically significant differences were noted (**b)**; The results of BrdU incorporation assay showed that mOECs derived from the MET group were characterized by suppressed replicative DNA synthesis (**c**). Similarly, the clonogenic potential (colony forming unit efficiency/CFU-E) of OECs expanded ex vivo from MET mice was lower in comparison to the cells derived from the CTRL group (**d**–**f**). The results are expressed as means ± SD. * *p* value < 0.05, ** *p* value < 0.01.

We determined metabolic ability, DNA synthesis and clonogenic potential of mOECs to investigate whether metformin administration in mice contributes to their ex vivo proliferation (Figure 3). The results indicated that mOECs derived from MET animals had impaired proliferative activity when compared with OECs from CTRL mice. Decreased proliferation was associated with reduced metabolic activity, lowered DNA synthesis, longer population doubling time and disturbed clonogenic potential. The analysis of literature shows that various factors can affect the proliferative status of OECs, such as the age of donor animal or species from which the tissue is collected [42,43]. Generally, the proliferative capacity of OECs in vitro is limited, which is associated with their mitotic quiescence upon dissociation [44]. It seems that the identification of factors that could promote their activity would be of great interest [24,44]. However, it was noted that prolonged mitogenic stimulation of both Schwann cells and OECs may cause spontaneous immortalization of these cells. It is assumed that the susceptibility of spontaneous immortalization of rodent OECs is associated with enhanced expression of GFAP and lowered expression of p75^NTR^ [45], which is consistent with our results. The proliferative activity of OECs may be enhanced by a combination of growth factors, for example, neuregulins, fibroblast growth factor 2 (FGF-2) and agents elevating the intracellular cAMP level, such as forskolin or dibutyryl-cAMP (dbcAMP) [46]. Anti-diabetic action of metformin also involves the reduction of cAMP synthesis [47], thus the observed effect may explain our results associated with decreased proliferative activity. Interestingly, it was also showed that p75^NTR^-positive cells from the olfactory mucosa, highly similar to olfactory bulb OECs both morphologically and antigenically, could proliferate longer than those from the olfactory bulb when cultured in identical conditions, and did not require the addition of exogenous growth factors [48]. Furthermore, it was also reported that the slower doubling time reflected a less mature phenotype [49]. Following this line, a lower proliferative activity, but a higher proportion of progenitor cells in the transplanted population, could be more beneficial for neuroregenerative medicine applications.

### 2.3. Metformin Administration May Ameliorate the Viability of mOECs

**Figure 4:**
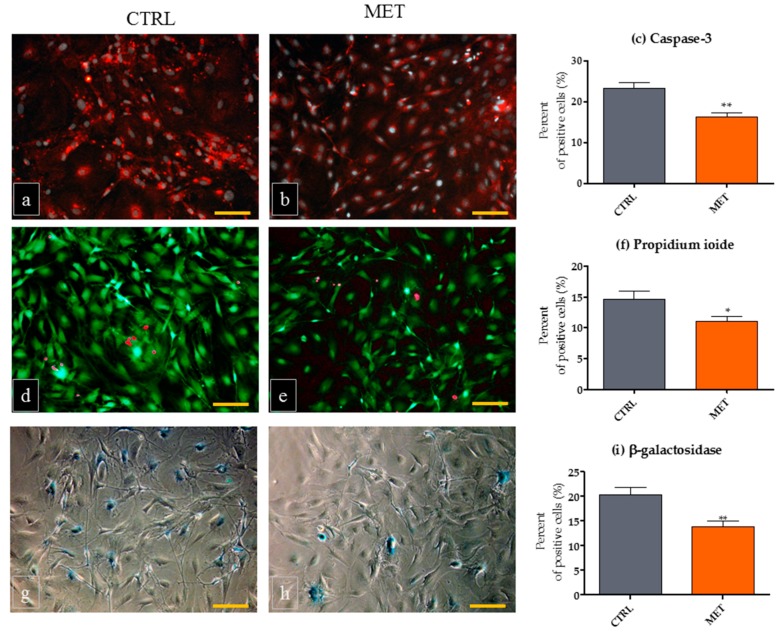
Viability of mouse olfactory ensheathing cells (mOECs) derived from animals from the control group (CTRL) and those receiving metformin (MET). OECs derived from MET mice and cultured ex vivo were characterized by the lowered expression of caspase 3 when compared to the mOECs from CTRL animals (**a**–**c**); The presence of dead cells was more prominent in OECs than in CTRL mice (**d**–**f**); Similarly, SA-βgal positive cells were more abundant in the CTRL mOEC cultures (**g**–**i**). Images of caspase-3 and calcein AM-propidium ioide reactions were captured using an epifluorescence microscope while β-gal cells were observed with a phase contrast microscope (scale bar = 250 µm). A minimum of three figures were analyzed with ImageJ to perform quantitative analysis. The results are expressed as means ± SD. * *p* value < 0.05, ** *p* value < 0.01.

We asked whether MET administration improved the ex vivo viability of mOECs; for this purpose, we determined the expression of caspase-3, evaluated the percentage of dead cells (propidium iodide-positive cells) and the activity of SA-βgal (Figure 4). The results indicated that metformin can promote survival of mOECs cultured ex vivo, which was associated with a decreased expression of caspase-3 and a lowered number of dead cells, when compared to mOECs from the CTRL group. The pro-survival action of metformin was described previously by Chang et al., who showed that metformin may inactivate caspase-3, known as a crucial mediator of apoptosis through its protease activity [13]. Additionally, we observed senolytic action of MET on mOECs, as a decrease of SA-β-gal activity, which is a reliable and sensitive marker for the detection of cellular senescence. The obtained results are consistent with the most recent observation of Chen et al. [19], who demonstrated anti-apoptotic and anti-senescence effects of metformin on nucleus pulposus cells. Metformin was shown to target senescent cells and a certain senescence-associated secretory phenotype (SASP) interfering with pro-inflammatory nuclear factor-κB signaling [50]. It was shown that metformin could exert an immunomodulatory effect by suppressing the production of inflammatory cytokines in senescent cells. Metformin inhibited the expression of IL-1b, IL-6, IL-8, i.e., cytokines that impair tissue homeostasis and promote chronic inflammation. Interestingly, this effect was not dependent on AMPK activation or even on the context of cellular senescence, which was clearly demonstrated by Moiseeva et al. Metformin inhibited NF-κB pathway, which was shown to be stimulated by lipopolysaccharide (LPS) in ampk-null fibroblasts and in macrophages [50]. These findings also highlight the potential application of MET in the prevention of neurodegenerative conditions.

### 2.4. Metformin Reduces the Expression of Oxidative Stress Markers in mOEC Cultures Derived from Animals Receiving MET

**Figure 5:**
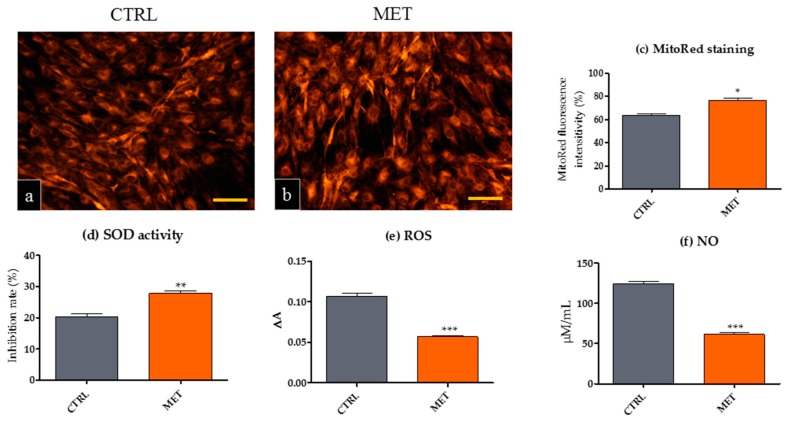
Visualization of mitochondria (**a**–**c**) and the assessment of SOD activity (**d**) and oxidative stress markers (**e**,**f**) in mOEC ex vivo cultures from CTRL and MET animals. The obtained data indicated that metformin administration decreased oxidative stress in OECs derived from MET mice. Additionally, it simultaneously improved antioxidative protection associated with increased SOD (superoxide dismutase) activity and lowered production of ROS (reactive oxygen species) and NO (nitric oxide). The results are expressed as means ± SD. * *p* value < 0.05, ** *p* value < 0.01; *** *p* value < 0.001.

We measured extracellular ROS and NO production, as well as the activity of SOD, to investigate whether the mechanism of senescent cell clearance induced in mOECs by MET administration was associated with the inhibition of oxidative stress markers. We also visualized active mitochondria with MitoRed staining (Figure 5). The results indicated that mOECs derived from MET animals launched adaptive responses that enhanced antioxidative defense mechanisms against reactive oxygen species (ROS and NO) associated with increased SOD expression and improved mitochondrial activity. This observation is consistent with previous findings, showing that metformin profoundly attenuates the production of ROS in AMPKα^+/+^ and AMPKα^−/−^ mouse embryonic fibroblasts [51]. We have previously shown that metformin reduces the accumulation of oxidative stress markers in mouse adipose-derived stromal cells isolated from animals receiving metformin [52]. Hou et al. [53] proposed a possible mechanism, wherein metformin reduces intracellular ROS levels; they emphasized the role of AMPK-FOXO3 pathway activation by increased expression of antioxidant thioredoxin (Trx). Oxidative stress mechanism becomes more visible during the process of aging, making neurons more sensitive to degeneration and development of neurodegenerative disorders. The decrease of ROS induced by MET could reduce DNA damage, and thus positively affects self-renewal and neurogenesis [54]. Metformin’s ability to directly scavenge ROS was also related to the increased expression of several mitochondrial genes with simultaneous preservation of mitochondrial complex I, II and III activity. The neuroprotective role of metformin associated with decreased ROS activity was also demonstrated in the studies using a model of 1-methyl-4-phenyl-1,2,3,6-tetrahydropyridine (MPTP)-induced Parkinsonian mice; they also addressed the neuroprotective effect of metformin through the enhanced expression of BDNF in substantia nigra [55]. This observation leads us to the next question, i.e., whether metformin affects the expression of BDNF in mOBCs from the MET group.

### 2.5. Metformin Increases Circulating Levels of BDNF and Affects Downstream Genes in the BDNF Pathway

**Figure 6:**
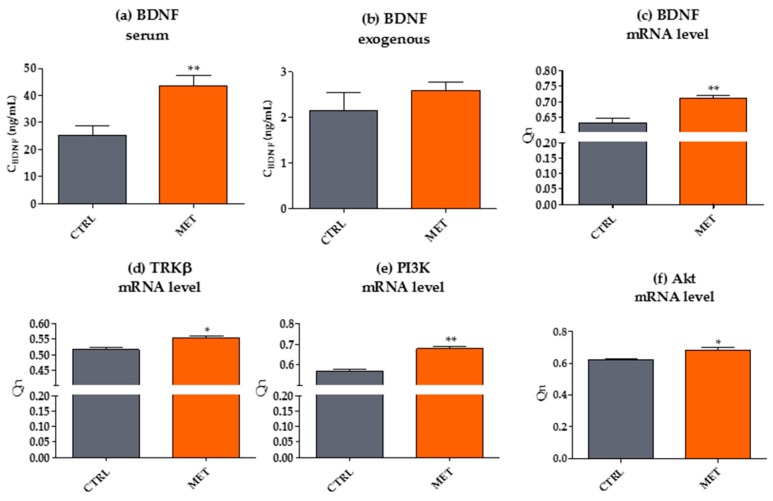

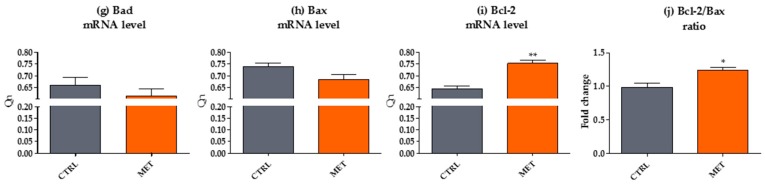
The influence of metformin administration on BDNF levels and expression of its signaling molecules. Quantitative analysis of BDNF concentration was performed using a specific ELISA assay. Circulating levels of BDNF were measured in the serum of animals, both in CTRL and MET groups (**a**) and the amount of secreted BDNF was also determined (**b**). Transcript levels were determined using qRT-PCR (**c**–**j**). A detailed description of the relationship between downstream target genes of the BDNF signaling pathway and OEC survival can be found in the text. The results are expressed as means ± SD. * *p* value < 0.05, ** *p* value < 0.01.

To address our last question, we determined the serum level of BDNF and analyzed the expression of genes involved in the BDNF pathway in mOEC in both groups of experimental animals (Figure 6). The results indicated that mice receiving metformin at a dose of 2.8 mg/day had a significantly increased level of serum BDNF. Our results are consistent with observations of Patil et al. [55], who reported that, in addition to antioxidant properties, metformin showed neuroprotective activity and neurotrophic potential by enhancing the expression of BDNF in the substantia nigra. Furthermore, a recent study of Wang et al. [30] implied that BDNF played a crucial role in the migration of OECs. It was postulated that BDNF secreted by injured tissues and/or OECs could promote the migration of transplanted OECs, thereby affecting the regeneration-promoting ability of OECs [30]. We were interested in whether MET administration influenced secretory activity of mOECs ex vivo. Our results revealed that mOECs from the MET group produced higher concentrations of BDNF; however, the observed differences were not statistically significant. The role of paracrine BDNF derived from OECs was showed previously by Sasaki et al. [56], who indicated that BDNF expression was increased after OEC transplantations in a spinal cord injury site. This observation, consistent with the results of Wang et al. [30], highlights the role of OECs as cells that have distant neuroprotective effects, associated with secretion of trophic substances and/or activation of endogenous neurotrophic secretion. In this light, secretion of BDNF by OECs promoted by metformin can be potentially utilized in the neurorescue or restorative treatment of neurodegenerative disorders [55,57]. BDNF is considered as one of the most promising neurotrophic factors due to its crucial role in the development and survival of neurons. It is well known that BDNF exerts its biological effect by binding to transmembrane receptors of two different classes, i.e., p75^NTR^ and the tyrosine kinase receptor (TrkB) [57]. Wang et al. showed that the role of BDNF in OEC biology is mediated by TrkB. The activation of TrkB by BDNF leads to an enhanced PI3K/Akt signaling [58]. Akt, activated by PI3K, promotes neuronal survival, coordinating the effects of growth factors and neural activity throughout the nervous system [59]. Moreover, Akt was found to sequester pro-apoptotic proteins (namely BAD) in the cytoplasm away from their transcriptional targets [60]. Our results showed an increased transcription of BDNF mRNA in mOECs derived from MET animals. The transcript level of BDNF receptor (TrkB) and downstream genes (PI3K and Akt) was also upregulated in mOECs harvested from the MET group. We also found a trend regarding the decreased mRNA levels of pro-apoptotic BAD and BAX genes; however, the observed tendencies were not statistically significant. Simultaneously, Bcl-2 transcript levels were significantly increased in OECs isolated from MET mice. Our results are consistent with the study of Fatt et al. [61], who characterized metformin as an optimal preconditioning agent that could be used to improve neuroregenerative efficiency of progenitor cells, propagated ex vivo before transplantation for the treatment of brain injury and neurodegenerative diseases. For example, BDNF-induced TrkB receptor signaling was shown to be crucial in rescuing caspase-3-mediated cell death, specifically in Huntington mutant striatal cells [62]. Additionally, Gupta et al. [11] using a differentiated neuronal cell line submitted to chronic hyperinsulinemia (Neuro-2a, neuroblastoma cell line) showed that metformin may prevent amyloid β (Aβ) generation and tau protein hyperphosphorylation. This suggests that metformin could be also used for the treatment of Alzheimer’s disease. On the other hand, Imfeld et al. [34] indicated that prolonged usage of metformin is associated with a slightly higher risk of AD development. This stands in good agreement with the studies of Allard et al. [3], who showed that metformin may decrease expression of the antioxidant pathway regulator i.e., Nrf2. It seems that, due to a wealth of information regarding the neuroprotective or neurodegenerative function of metformin, we are still far from a clear consistent picture of its mechanism of action.

## 3. Materials and Methods

### 3.1. Experimental Animals

The experiments were conducted with the consent of the II Local Ethics Committee of Environmental and Life Science University (Decision no. 177/2010 of 15 November 2010). The experimental conditions of animal housing have been described in details previously [52,63]. In this study, we used twelve 4-week-old female mice (C57BL/6 strain) kept at 22 ± 0.2 °C, three per cage in an ultraclean facility on ventilated racks housed in the Animal Experimental Laboratory (Wroclaw Medical School, Norwida, Poland). During the eight-week experiment, a 12-h light-dark cycle was used. Additionally, the animals were fed with a standard diet containing 4.2% fat (Morawski, Labofeed H, Kcynia, Poland). Water and feed was administrated ad libitum. Mice were randomly divided into two groups i.e., control (*n* = 6) and experimental (*n* = 6). Experimental mice received metformin (Metformax 850; Teva Pharmaceuticals, Warszawa, Poland) in drinking water at concentration equal to 2.8 mg per day. The water was changed every two days. After the experiment, mice were euthanized using carbon dioxide. Olfactory bulbs were collected for OEC isolation.

### 3.2. Isolation and Culture of Mice Olfactory Ensheathing Glial Cells (mOECs)

Mouse OECs were isolated using a method established previously in a rat model [64]. Tissue samples were finely cut with surgical scissors and incubated for 10 min at 37 °C in 0.2% collagenase solution. Following collagenase digestion, the tissue was homogenized using syringe needles (18, 20, 22 G). The olfactory bulb homogenates were washed with Hank’s balanced salt solution (HBSS; Sigma Aldrich, Munich, Germany) and centrifuged for 5 min at 300× *g*. The resulting pellets were suspended in a complete growth medium (CGM) consisting of Dulbecco’s Modified Eagle’s Medium with Nutrient F-12 Ham and supplemented (Sigma Aldrich, Munich, Germany) with 10% fetal bovine serum (FBS, Sigma Aldrich, Munich, Germany). Additionally, a 2% antibiotic/antimycotic solution was added to CGM. Primary cultures of mOECs were maintained in the incubator with 5% CO_2_ and 95% humidity at 37 °C in T-25 flasks. CGM was not changed within first 72 h of mOEC cultures; after this time, media was changed every two to three days. Cultures of mOECs were passaged using trypsin solution (TrypLE™ Express, Thermo Fisher Scientific, Warszawa, Poland), when the cells reached about 80% confluence. The cells were passaged three times before using them in the experiments.

### 3.3. Analysis of mOEC Morphology

For the analysis, the cells (p. 3) were inoculated to 24-well plates at a density of 3 × 10^4^ cells per well. Microscopic observations were performed after six days of culture (144 h). Cell morphology was evaluated using phase contrast microscopy (PCM/Axio Observer A.1, Zeiss, Oberkochen, Germany). Visualization of cultures with epifluorescent microscopy (EpiFM/Axio Observer A.1, Zeiss, Oberkochen, Germany) required culture fixation in ice-cold 4% paraformaldehyde (PFA, Sigma Aldrich, Munich, Germany) for 15 min at room temperature. Cultures designated for scanning electron microscopy (SEM) were fixed with 2.5% glutaraldehyde for 1 h at room temperature.

The procedure of culture staining with 4′,6-diamidino-2-phenylindole (DAPI; 1:1000) and phalloidin (atto-565; 1:800) was performed according to the protocol described previously [65,66]. Both dyes were purchased from Sigma Aldrich (Munich, Germany). Documentation was performed with a digital camera (Cannon PowerShot A640, Canon, Warszawa, Poland). The obtained pictures were merged using ImageJ software (version 1.6.0, U. S. National Institutes of Health, Bethesda, MD, USA). Preparation of samples for SEM imaging has also been described in details elsewhere [64,66]. In the present experiment, the cultures were dehydrated in a graded ethanol series (from 50% to 100%, increasing 10% at each step). The samples were coated with gold particles using the 300-s program (Edwards, Scancoat six, HHV Ltd., Crawley, UK). Prepared specimens were imaged using a SE1 detector at 10 kV filament tension (SEM, Evo LS 15, Zeiss, Oberkochen, Germany) and 1000× and 2000× magnification.

### 3.4. Phenotype of Mice OECs

For immunophenotyping, mOEC cultures (p. 3) were inoculated at a density of 3 × 10^4^ on 24-well plates. After six days of culture, the cells were fixed with PFA and incubated in 1% bovine serum albumin/10% normal goat serum/0.3 M glycine in 0.1% PBS-Tween for 1 h to permeabilize the cells and block nonspecific protein–protein interactions. The cells were then incubated overnight with primary antibodies, i.e., rabbit antiglial fibrillary acidic protein (anti-GFAP, Abcam, Cambridge, UK) and rabbit p75 nerve growth factor receptor (NGFR, Bioss Antibodies, Gentaur Poland Sp. z o.o., Sopot, Poland) at 4 °C. The secondary antibody used for the reaction was goat anti-rabbit conjugated with Alexa Fluor 488 (Abcam, Cambridge, UK). Cultures were incubated with secondary antibody for 1 h at 4 °C. Primary antibodies were diluted to a concentration of 1:100, whereas secondary antibodies were diluted to a concentration of 1:1000. Cell nuclei were counterstained with DAPI (1:1000) for 5 min at room temperature. Protocols used for phenotypic characterization of cells have been published previously [64,67,68]. Images were analyzed using ImageJ and Pixel Counter plugin (version 1.6.0, U. S. National Institutes of Health, Bethesda, MD, USA) [69,70].

### 3.5. Analysis of mOEC Proliferation

Comprehensive assays were performed in order to determine the proliferative potential of mOECs. Metabolic activity of the cells was evaluated using a commercial resazurin-based assay (Alamar Blue, Sigma Aldrich, Munich, Germany); DNA synthesis was determined with a bromodeoxyuridine (BrdU) assay (Abcam, Cambridge, UK), while clonogenic potential was evaluated with a colony forming unit (CFU) assay. The procedures were performed accordingly to the protocols established previously in multipotent stromal cells [49,65,71]. To analyze metabolic activity, the cells were inoculated to 24-well plates with a density equal to 3 × 10^4^. Metabolic activity of mOECs was monitored after 24, 72 and 144 h of culture in vitro. For this purpose, the cultures were incubated in a medium containing 10% Alamar Blue for two hours at predetermined time points. The absorbance of the supernatants was measured spectrophotometrically (BMG LABTECH, Ortenberg, Germany) at a wavelength of 600 nm for resazurin and 690 nm as a reference wavelength. The metabolic activity was expressed as ΔΔA value including absorbance of blank samples. Additionally, the population doubling time (PDT) of mOECs derived from control (CTRL) and experimental (fed with metformin, MET) mice was determined using an algorithm proposed previously by Heuer et al. [72] and supported by the population doubling time online calculator (Cell Calculator ++) [73]. The number of mOECs was estimated based on the cell growth curve determined during the test. The analysis of DNA synthesis was performed with the BrdU Cell Proliferation ELISA Kit (Abcam, Cambridge, UK) after 144 h of culture. For BrdU incorporation assay, mOECs were inoculated to a 96-well plate in an amount of 2 × 10^4^ into each well. Colorimetric detection of specific reaction was performed with a spectrophotometer microplate reader (BMG LABTECH, Ortenberg, Germany) at a wavelength of 450/550 nm. BrdU ELISA test sensitivity was <40 cells/well. For the clonogenic assays, mOECs were inoculated into 6-well plates at a density of 1 × 10^3^. After ten days of culture, the cells were fixed with 4% PFA and visualized with pararosaniline staining. Colonies formed by more than 30 cells were considered as a colony-forming unit (CFU). Colony forming efficiency was calculated using the following formula: $\frac{number\;of\;colonies\;>\;30\;cells\;}{number\;of\;inoculated\;cells\;}\;\times 100\%$. All experimental cultures were performed in triplicate.

### 3.6. Analysis of mOEC viability

Specific staining were performed in order to determine the effect of MET administration on mOEC viability. Detection of caspase-3 was performed using anti-caspase-3 polyclonal active antibody produced in rabbit (Sigma Aldrich, Munich, Germany). Immunostaining procedure was performed as described above (Section 3.4), except that the secondary antibody was goat anti-Rabbit IgG—Atto 594 (Sigma Aldrich, Munich, Germany). The amount of viable and dead cells was evaluated with Cellstain Double Staining Kit (Sigma Aldrich, Munich, Germany), according to the manufacturer’s instructions. In the reaction, viable cells were stained with Calcein-AM (acetoxymethyl) and emitted green fluorescence, whereas dead cells’ nuclei were stained orange with propidium iodide. Moreover, to identify the presence of β-galactosidase-associated senescence, the cells were stained using Senescence Cells Histochemical Staining Kit, according to the manufacturer’s protocol. Stained mOEC cultures were observed under an inverted microscope (Axio Observer A.1, Zeiss, Oberkochen, Germany) and analyzed with an ImageJ Pixel Counter plugin [69,70].

### 3.7. Visualization of Mitochondria and Determination of Oxidative Stress Factors in mOEC Cultures

In order to visualize the mitochondria, mOEC cultures were incubated with MitoRed dye (1:1000) in 37 °C for 30 min in CO_2_ incubator, according to the protocol of the manufacturer. For imaging with an epifluorescence microscope (Axio Observer A.1, Zeiss, Oberkochen, Germany), the cells were stained with 4% PFA. The intensity of fluorescent signal derived from mitochondria was analyzed using ImageJ Pixel Counter plugin [69,70]. Oxidative stress factors were determined in the supernatants collected after 144 h of mOEC cultures derived from MET and CTRL animals. Intracellular reactive oxygen species (ROS) were measured using H2DCF-DA solution (Thermo Fisher Scientific, Warszawa, Poland), while superoxide dismutase (SOD) was determined using a commercially available SOD determination kit (Sigma Aldrich, Munich, Germany). Nitric oxide (NO) activity was measured using Griess reagent kit (Thermo Fisher Scientific, Warszawa, Poland). The formation of oxidative stress biomarkers was evaluated spectrophotometrically with microplate reader (BMG LABTECH, Ortenberg, Germany). The reactions were performed accordingly to the manufacturers’ protocols. Each experiment was performed three times.

### 3.8. Analysis of mRNA for BDNF and Its Downstream Target Genes

After 144 h, the cultures of mOECs derived from CTRL and MET animals were homogenized using TRI Reagent^®^ (Sigma Aldrich, Munich, Germany). Subsequently, total RNA was isolated using the single-step method described by Chomczynski and Sacchi [74]. The quantity and quality of specimens were evaluated using a spectrophotometer (WPA Biowave II, Cambridge, UK). Total RNA (500 ng) was used to transcribe cDNA using PrimeScript™ RT reagent Kit with gDNA Eraser (Takara Bio Europe, Saint-Germain-en-Laye, France). Simultaneous total RNA purification and cDNA synthesis was performed on T100 Thermal Cycler (Bio-Rad, Hercules, CA, USA), according to the protocols supplied by the producer of the kit. The total volume of PCR was 20 µL, while cDNA 2 µL. The concentration of primers in the reaction mixture was 0.5 µM. The list of primers used in the reaction and their characteristics are presented in Table 1. Amplification of desired products was performed using SensiFast SYBR & Fluorescein Kit (Bioline Reagents Limited, London, UK) on a CFX Connect Real-Time PCR Detection System (Bio-Rad, Hercules, CA, USA). The following cycling conditions were applied in the reaction: 95 °C for 2 min, followed by 50 cycles at 95 °C for 30 s, annealing for 30 s, and elongation at 72 °C for 30 s with a single fluorescence measurement. All reactions were performed in three repetitions. The specificity of PCR products was determined by analyzing the dissociation curve of the amplicons. A melting curve was performed using a gradient program of the range from 65 to 95 °C at a heating rate of 0.2 °C/s and continuous fluorescence measurements. The value of the threshold cycle (*C*_t_) was used to calculate the fold change in relation to the expression of the housekeeping gene, i.e., β-actin (ACTB), as described previously [63].

### 3.9. Determination of BDNF Protein Levels—Secretory Activity of mOECs and Circulating Level of the Protein

BDNF concentrations in the serum and supernatants were analyzed after 144 h of mOEC propagation using an enzyme-linked immunosorbent assay (EIAab ELISA kit, Biokom, Warszawa, Poland), characterized by the detection range of 0.156–10 ng/mL. Mice sera were five-fold diluted for the analysis, while the supernatants remained undiluted. All tested samples and standards were measured in triplicate. Optical density was determined immediately after reactions at 450 nm wavelength using a microplate reader (BMG LABTECH, Ortenberg, Germany). The results were analyzed by comparing BDNF concentration in the experimental samples with calibration curve values.

### 3.10. Statistical Analysis

All experiments were performed at least in three replicates. Statistical analysis was performed using GraphPad Prism 5 software (version, Manufacturer, La Jolla, CA, USA). Differences between groups were determined by Student’s *t*-test or Mann–Whitney *U* test and two-way ANOVA (analysis of data obtained in Alamar Blue assay). Differences with a probability of *p* < 0.05 were considered significant.

## 4. Conclusions

Our study demonstrated that metformin administration may improve physiological activity of olfactory ensheathing cells ex vivo. In our model, metformin demonstrated senolytic, antioxidant and anti-apoptotic activity on OECs, but also stimulated an increased release of BDNF to the serum. In this context, metformin administration could help to obtain autologous OECs characterized with high cellular activity and maintaining great regenerative potential. However, due to the wealth of information regarding the neuroprotective or neurodegenerative functions of metformin, we are still far from a clear and consistent picture of its mechanism of action. The neuroprotective effects of metformin along with the regenerative potential of autologous OECs transplants could be determined deeply, for example, using the rodent model of spinal cord repair. Moreover, we are interested if the OECs treated in vitro with metformin will also be characterized by the high viability associated with cellular homeostasis in terms of oxidative status and increase in BDNF production. In light of this, metformin could be used as an agent reducing oxidative stress in OEC cultures before transplantation for central nervous system repair. Antioxidant and senolytic properties of metformin could be used to treat not only OECs, but other progenitor cells where long-term cultures and multiple passages are required to obtain a sufficient number of cells for transplantation.

## Figures and Tables

**Table 1:** Primer sequences used for the detection of selected genes.

Gene	Abbreviation	Primer	Sequence 5′–3′	Loci	Aplicon Lenght (bp)	Accesion No.
Brain derived neurotrophic factor	BDNF	F	GCCGCAAACATGTCTATGAGGGTT	670–693	174	NM_001316310.1
		R	TTGGCCTTTGGATACCGGGACTTT	843–820		
Tropomyosin receptor kinase B	TrkB/NTRK2	F	GCGAACCTGCAGATACCCAAT	1306–1326	148	XM_006517152.2
		R	CCAAATTCCCAACGTCCCA	1453–1435		
B cell leukemia/lymphoma 2	Bcl-2	F	ATCGCCCTGTGGATGACTGAG	1918–1938	129	NM_009741.5
		R	CAGCCAGGAGAAATCAAACAGAGG	2046–2023		
Bcl-2-associated death promoter	Bad	F	ACATTCATCAGCAGGGACGG	199–218	115	NM_001285453.1
		R	ATCCCTTCATCCTCCTCGGT	313–294		
Bcl-2-associated X protein	Bax	F	TGCTAGCAAACTGGTGCTCA	476–495	113	XM_011250780.1
		R	CTTGGATCCAGACAAGCAGC	588–569		
RAC-γ serine/threonine-protein kinase	Akt3	F	ATCCCCTCAACAACTTCTCAGT	450–471	156	XM_011238805.1
		R	CTTCCGTCCACTCTTCTCTTTC	605–584		
Phosphatidylinositol-4,5-bisphosphate 3-kinase	PI3K	F	CTCTCCTGTGCTGGCTACTGT	2932–2952	157	XM_006536015.2
		R	GCTCTCGGTTGATTCCAAACT	3088–3068		
β-actin	ACTB	F	CCTGAGGCTCTTTTCCAGCC	881–900	110	NM_007393.5
		R	TAGAGGTCTTTACGGATGTCAACGT	990–966		
